# Deconstructing Brain Waves: Background, Cue, and Response

**DOI:** 10.1371/journal.pbio.0020180

**Published:** 2004-06-15

Light waves from an awaited signal—a white circle—arrive at the subject's eye; within a fraction of a second, the subject's thumb presses a button. Between eye and thumb lies the central nervous system, its feats of perception, integration, and response largely opaque to scientific scrutiny. Imaging techniques like magnetic resonance imaging can detail brain anatomy but can only broadly show changes in activity levels occurring over seconds—indirect echoes of brain function. Electrodes stuck to the scalp record coordinated neuronal symphonies, and wires inserted among neurons can capture the single-cell firing patterns of the individual instruments of the neural orchestra. But how these electrical signals map to information processing within and across neural circuits remains blurry. A new analysis sharpens the focus by separating individual brain wave patterns, measured from multiple sites across the scalp, into nine distinct process classes, each centered in an anatomically relevant brain area and producing predictable patterns as human subjects receive visual cues and produce responses.[Fig pbio-0020180-g001]

**Figure pbio-0020180-g001:**
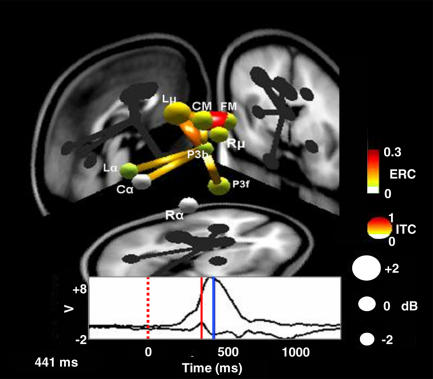
Schematic representation of the source and strength of task-related EEG signals. The animation can be accessed online at http://dx.doi.org/10.1371/journal.pbio.0020176.v001

Scalp electroencephalograms (EEGs) are dominated by waves of synchronized neuronal activity at specific frequencies. Decades of research have associated wave patterns recorded at different scalp regions with different states of alertness—attending, drowsy, sleeping, or comatose; eyes open or closed—and gross abnormalities, such as seizure, brain damage, and tumor. In order to separate EEG responses to specific events from background, state-related activity, researchers repeat an experiment like the button-press exercise tens or hundreds of times and average the EEG across trials. By averaging out background activity, this technique reveals a characteristic waveform, called an event-related potential (ERP). It differs by electrode location, but often contains a large positive wave that peaks 300 milliseconds or more after an awaited visual cue.

In the current paper, Scott Makeig et al. argue that ERP averaging removes important information about ongoing processes and their interactions with event-related responses. Instead of averaging multiple recordings from each of 31 electrode sites, the authors applied an algorithm that seeks independent signal sources contributing to the individual tracings. The researchers measured signal source activities by the frequency and phase of wave patterns and source locations by comparing signal strength and polarity at different electrodes. Altogether, the researchers identified nine classes of maximally independent sources, each having similar locations and activities across subjects. The results dovetail neatly with prior anatomical and functional observations.

This analysis demonstrates that average waveforms identified in ERP studies probably sum multiple, separate processes from several brain regions. In particular, the large positive ERP seen 300 milliseconds or more after a visual cue reflects different waveforms from frontal, parietal, and occipital cortex—areas involved in task planning, spatial relationships and movement, and visual processing, respectively. In addition, this study showed a two-cycle burst of activity in the 4–8 (theta) frequency band after button presses—another common ERP feature. The theta activity was coordinated across several signal sources, and localized to areas associated with planning and motor control. Notably, the planning component seemed to lead the motor signal. Suppression or resynchronization of several EEG processes followed the visual cue or button press. The authors theorize that such coordination might influence the speed or impact of communication between brain areas and help retune attention after significant events.

Using this approach in more subjects, and under differing conditions, could provide an unprecedented glimpse of how the brain translates perception and planning into action. The results suggest that EEG data contain an untapped richness of information that could give researchers and clinicians a new window into thought in action.

